# Correction to: Mild traumatic brain injury induces microvascular injury and accelerates Alzheimer-like pathogenesis in mice

**DOI:** 10.1186/s40478-023-01719-2

**Published:** 2024-02-15

**Authors:** Yingxi Wu, Haijian Wu, Jianxiong Zeng, Brock Pluimer, Shirley Dong, Xiaochun Xie, Xinying Guo, Tenghuan Ge, Xinyan Liang, Sudi Feng, Youzhen Yan, Jian‑Fu Chen, Naomi Sta Maria, Qingyi Ma, Fernando Gomez‑Pinilla, Zhen Zhao

**Affiliations:** 1 Center for Neurodegeneration and Regeneration, Zilkha Neurogenetic Institute, Room: 241, 1501 San Pablo Street, 90033 Los Angeles, CA USA; 2 Department of Physiology and Biophysics, Keck School of Medicine, University of Southern California, 90033 Los Angeles, CA USA; 3 Department of Neurosurgery, Second Affiliated Hospital, School of Medicine, Zhejiang University, 310009 Hangzhou, Zhejiang China; 4 Neuroscience Graduate Program, Keck School of Medicine, University of Southern California, 90033 Los Angeles, CA USA; 5 Center for Craniofacial Molecular Biology, Herman Ostrow School of Dentistry, University of Southern California, 90033 Los Angeles, CA USA; 6 Lawrence D. Longo, MD Center for Perinatal Biology, Division of Pharmacology, Department of Basic Sciences, Loma Linda University School of Medicine, 92350 Loma Linda, CA USA; 7 Brain Injury Research Center, Department of Neurosurgery, University of California, Los Angeles, 90095 Los Angeles, CA USA

**Correction to:** ***Acta neuropathol commun*** **9, 74(2021)**

10.1186/s40478-021-01178-7

Following publication of the original article [1], the authors identified errors in Fig. 4; the two images of the dentate gyrus (DG) in Fig. 4a, and two images of CA1 in Fig. 4b. We have determined that there is a high degree of similarities between these images. After we located the original confocal scans in our data storage, we believe they were not intentional duplications, but rather a misplacement of scans in the incorrect folders. The two scans in mTBI groups were taken on later dates, but mistakenly saved to the folder belongs to the Sham group. During the manuscript preparation, they were mistakenly used, since they represent similar brain structures as the rest images in the mTBI group. As the images were not exactly the same, this mistake was not detected by us during the manuscript preparation, or by the reviewers during the reviewing process, and end up being published. The authors have determined that this error in the representative images did not affect the data analysis, nor had impact on the scientific conclusions of the paper in any way. Unfortunately, the authors did not have the capacity at that time to run image analysis with capable software; otherwise, such human errors could have been prevented.

The correct figure and caption is given hereafter.

The incorrect Fig. 4:

**Fig. 4:**
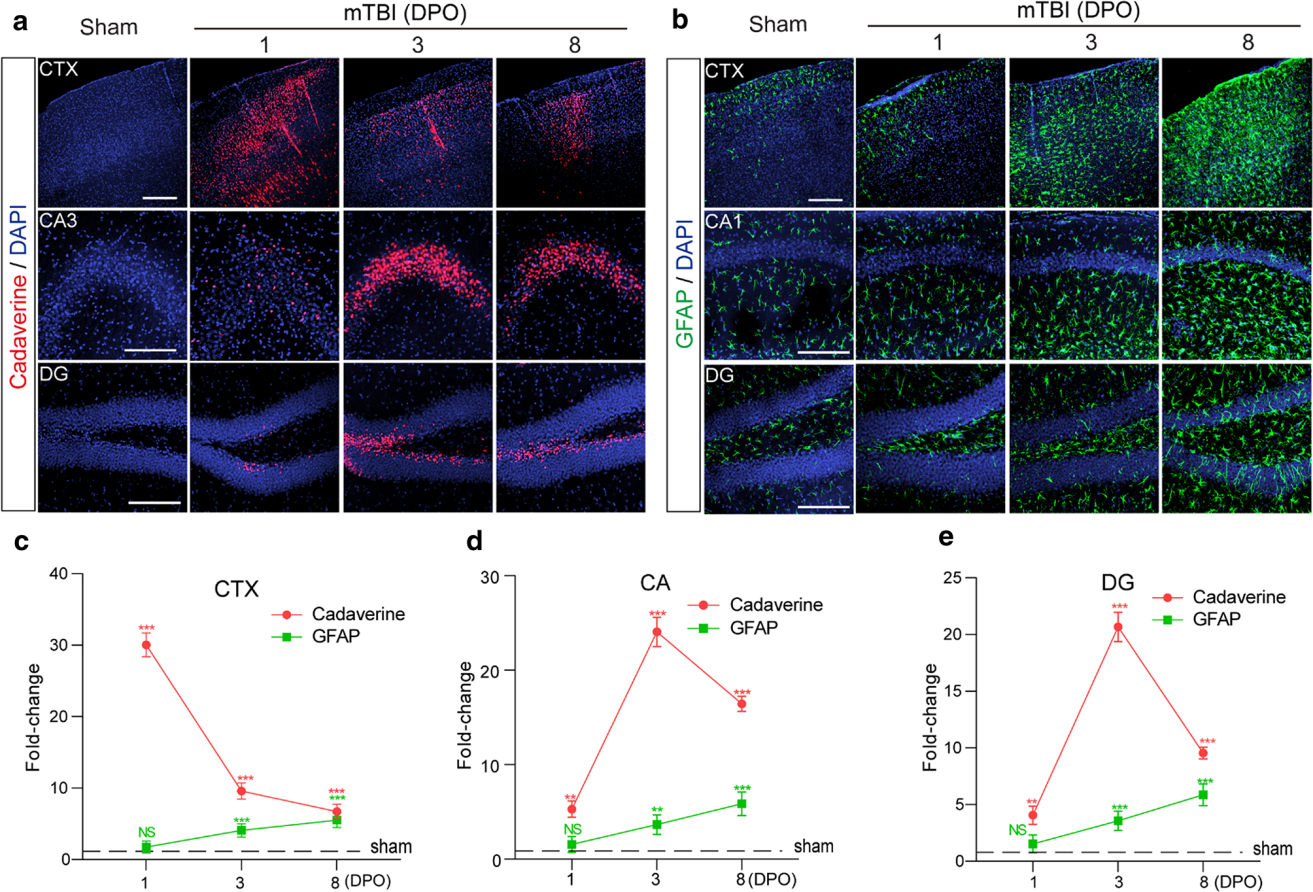
The time courses of BBB dysfunction and astrogliosis after mTBI in mice. **a** Representative confocal microscope images showing the extravasation of intravenously administrated Alexa-555 cadaverine (red) in cortex (CTX), Cornu Ammonis 3 (CA3) and dentate gyrus (DG) area 1-day, 3- and 8- days post operation (DPO). Scale bar = 100 μm. **b** Representative confocal microscope images showing GFAP-positive astrocytes (green) in cortex (CTX), CA1 and dentate gyrus (DG) area 1-day, 3- and 8- days post operation (DPO). Scale bar = 100 μm. **c–e** Quantification for the fold changes for cadaverine intensity (*n* = 5 mice per time point) and GFAP positive cells (*n* = 5 mice per time point) in CTX area, CA area and DG area 1-day, 3- and 8- days post operation (DPO). In **c–e**, data are presented as mean ± SD; ***, *P* < 0.001; **, *P* < 0.01 NS, non-significant (*P* > 0.05) compare to sham group, one-way ANOVA followed by Bonferroni’s post-hoc tests. Dash lines indicate the sham-operated group.

The correct Fig. 4:

**Fig. 4:**
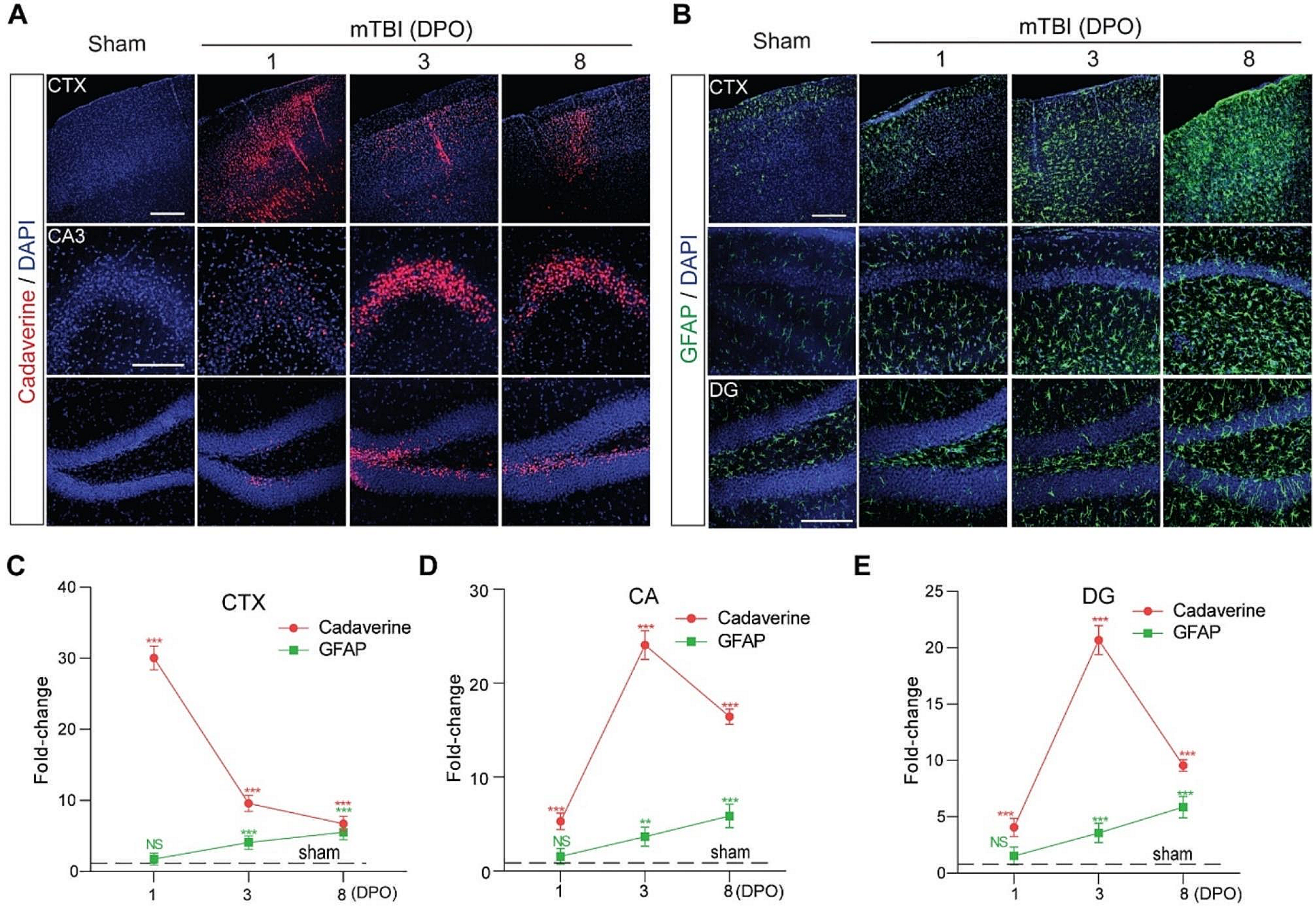
The time courses of BBB dysfunction and astrogliosis after mTBI in mice. **a** Representative confocal microscope images showing the extravasation of intravenously administrated Alexa-555 cadaverine (red) in cortex (CTX), Cornu Ammonis 3 (CA3) and dentate gyrus (DG) area 1-day, 3- and 8- days post operation (DPO). Scale bar = 100 μm. **b** Representative confocal microscope images showing GFAP-positive astrocytes (green) in cortex (CTX), CA1 and dentate gyrus (DG) area 1-day, 3- and 8- days post operation (DPO). Scale bar = 100 μm. **c–e** Quantification for the fold changes for cadaverine intensity (*n* = 5 mice per time point) and GFAP positive cells (*n* = 5 mice per time point) in CTX area, CA area and DG area 1-day, 3- and 8- days post operation (DPO). In **c–e**, data are presented as mean ± SD; ***, *P* < 0.001; **, *P* < 0.01 NS, non-significant (*P* > 0.05) compare to sham group, one-way ANOVA followed by Bonferroni’s post-hoc tests. Dash lines indicate the sham-operated group

Figure 4 has been updated above and the original article [1] has been corrected.

## References

[1] Wu Y, Wu H, Zeng J (2021). Mild traumatic brain injury induces microvascular injury and accelerates Alzheimer-like pathogenesis in mice. acta Neuropathol Commun.

