# Supplementary material for: Mild traumatic brain injury induces microvascular injury and accelerates Alzheimer-like pathogenesis in mice
Source: Acta Neuropathol Commun. 2021 Apr 23;9:74. doi: 10.1186/s40478-021-01178-7 (PMC8063402; doi:10.1186/s40478-021-01178-7)
Supplement: Supplementary file 1 — Additional file 1: Supplementary figures. Supplementary Fig. 1 Traumatic brain injury models with controlled cortical impact in mice. (A) The timeline for the establishment of the TBI mouse model and the other experimental procedures. (B) Brain precision impactor device (RWD Life Science) to establish mTBI mouse model. (C-D) The experimental parameters used in the establishment of TBI mouse models. Supplementary Fig. 2 Neuropathological changes 3 days after sTBI or mTBI in mouse model. (A) Cerebral edema measured by brain water content. The injury significantly induced the development of cerebral edema at 24 hours post-injury in sTBI model compared to mTBI model. n = 8 mice per group, Data are presented as Mean ± SD; ***, P < 0.001; NS, non-significant (P > 0.05), one-way ANOVA followed by Bonferroni′s post-hoc tests. (B) Representative confocal images showing NeuN (green) staining and TUNEL assay (red) for apoptotic neuronal death in the impacted ipsilateral area after sTBI and mTBI, or sham-operation. Scale bar, 50 μm. (C) Quantification of the percentage of TUNEL-positive neuronal death in sTBI (n = 6 mice) or mTBI (n = 6 mice). Data are presented as Mean ± SD; *** P < 0.001 by Student′s t-test. Dash line indicates an average value from sham-operated group (n = 3 mice). (D-E) Representative immunoblots (D) and quantification (E) of Synapsin I from the cortex in the injury ipsilateral side of sham-operated, mTBI and sTBI mice. β-Actin: loading control. Data are presented as Mean ± SD; n = 3 mice per group respectively; ***, P < 0.001; **, P < 0.01; one-way ANOVA followed by Bonferroni′s post-hoc tests. Supplementary Fig. 3 Neuroinflammatory changes 3 days after sTBI or mTBI in mouse model. (A) Representative images of fluorescence immunostaining for Iba1 (gray) and DAPI (blue), boxed regions show an activated and phagocytotic microglia (a1, a3) in the impacted ipsilateral side, and ramified microglia at resting state (a2, a4) in the contralateral side. Scale bar i [file 40478_2021_1178_MOESM1_ESM.docx]

**SUPPLEMENTARY DATA**


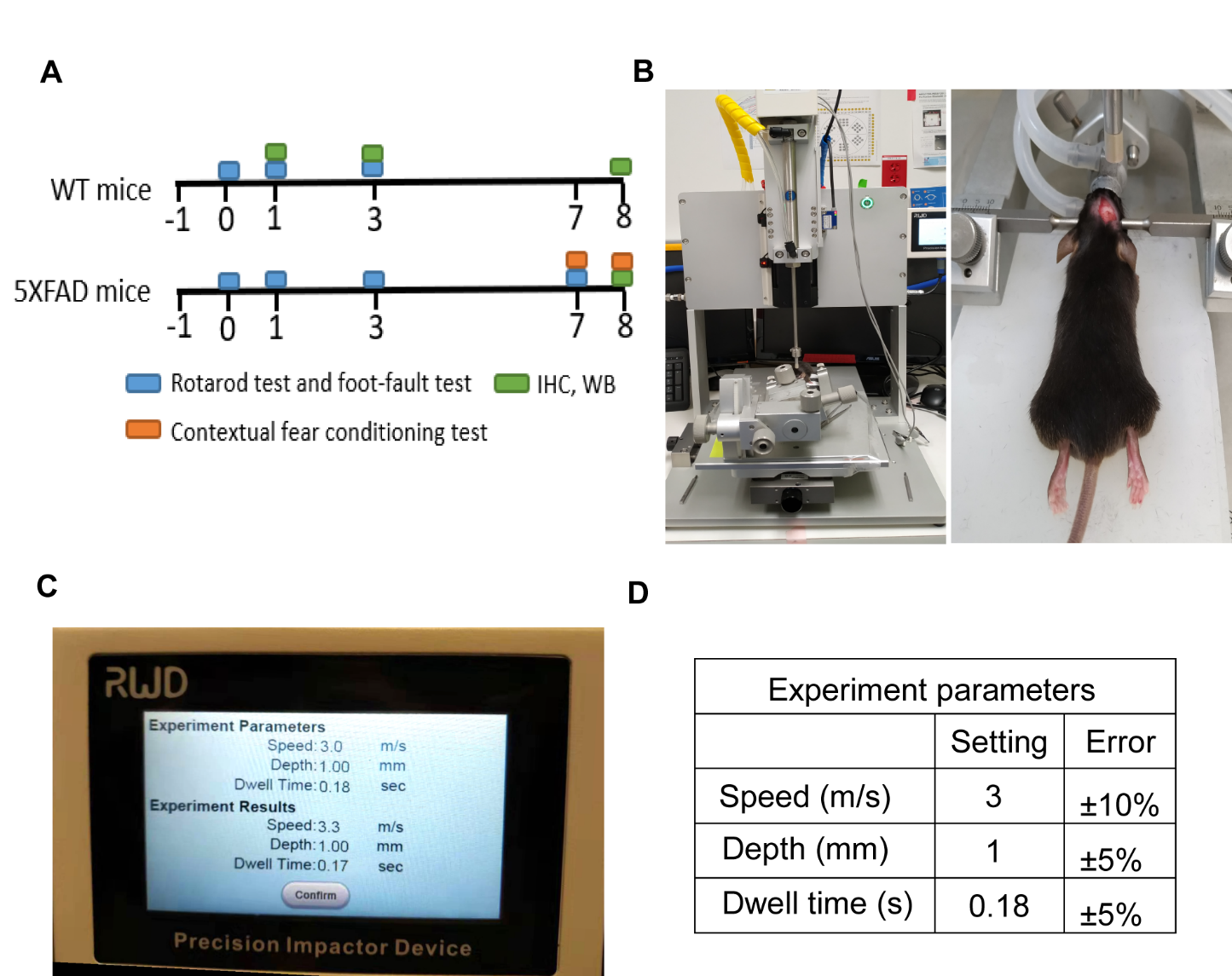


**Supplementary Fig. 1**. Traumatic brain injury models with controlled cortical impact in mice. (**A**) The timeline for the establishment of the TBI mouse model and the other experimental procedures. (**B**) Brain precision impactor device (RWD Life Science) to establish mTBI mouse model. (**C**-**D**) The experimental parameters used in the establishment of TBI mouse models.


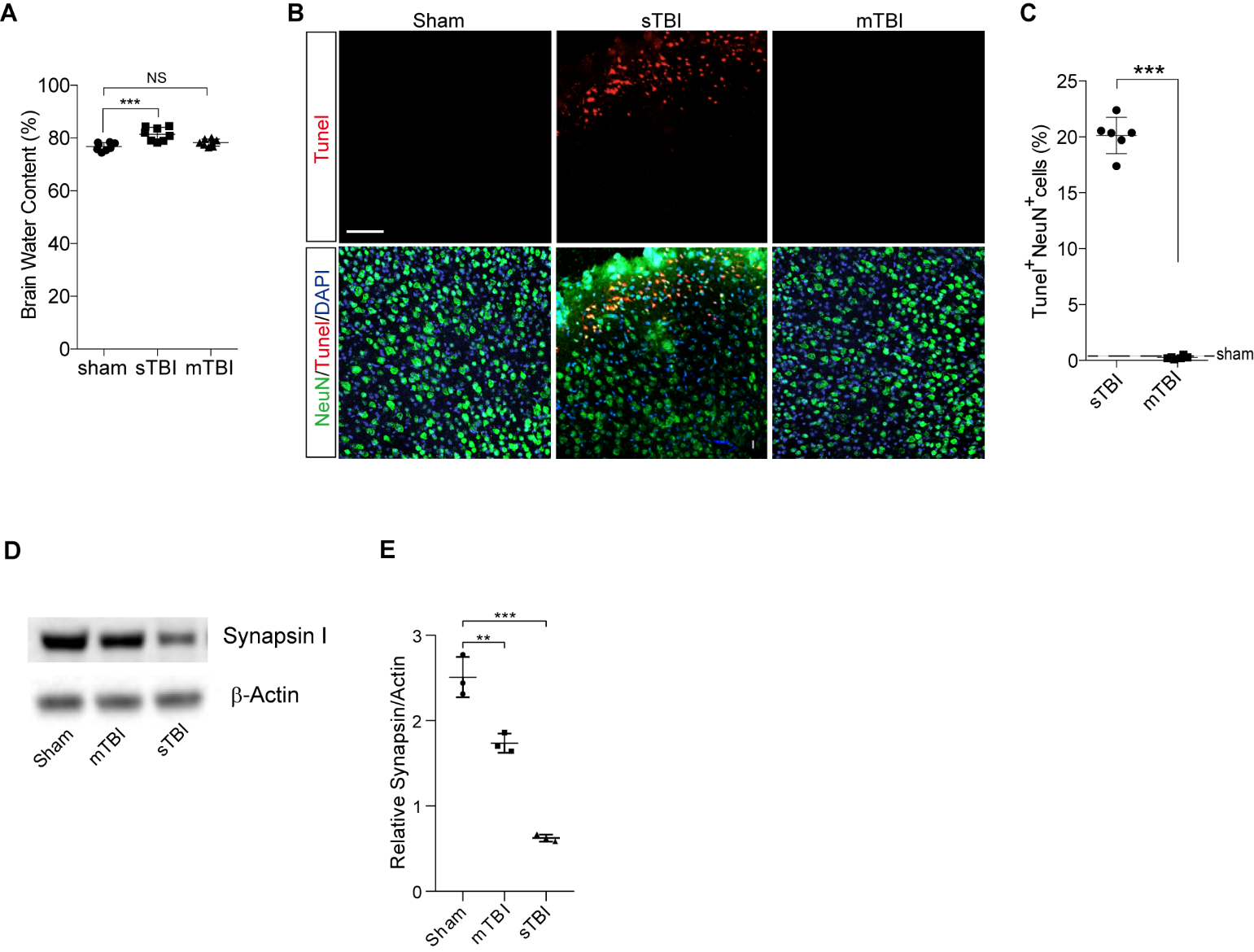


**Supplementary Fig. 2**. **Neuropathological changes 3 days after sTBI or mTBI in mouse model**. (**A**) Cerebral edema measured by brain water content. The injury significantly induced the development of cerebral edema at 24 hours post-injury in sTBI model compared to mTBI model. *n = 8* mice per group, Data are presented as Mean ± SD; ***, *P* < 0.001; NS, non-significant (*P* > 0.05), one-way ANOVA followed by Bonferroni’s post-hoc tests. (**B**) Representative confocal images showing NeuN (green) staining and TUNEL assay (red) for apoptotic neuronal death in the impacted ipsilateral area after sTBI and mTBI, or sham-operation. Scale bar, 50 μm. (**C**) Quantification of the percentage of TUNEL-positive neuronal death in sTBI (*n = 6* mice) or mTBI (*n = 6* mice). Data are presented as Mean ± SD; *** *P* < 0.001 by Student’s t-test. Dash line indicates an average value from sham-operated group (*n = 3* mice). (**D**-**E**) Representative immunoblots (**D**) and quantification (**E**) of Synapsin I from the cortex in the injury ipsilateral side of sham-operated, mTBI and sTBI mice. β-Actin: loading control. Data are presented as Mean ± SD; *n = 3* mice per group respectively; ***, *P* < 0.001; **, *P* < 0.01; one-way ANOVA followed by Bonferroni’s post-hoc tests.


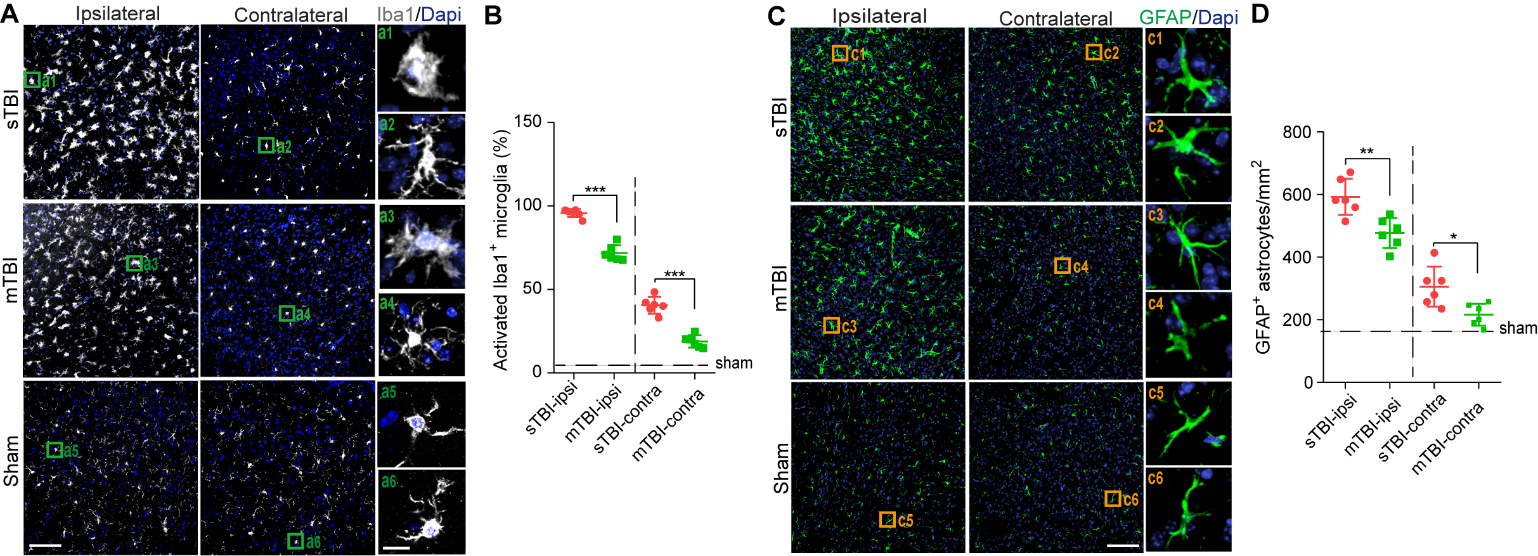


**Supplementary Fig. 3**. **Neuroinflammatory changes 3 days after sTBI or mTBI in mouse model**. (**A**) Representative images of fluorescence immunostaining for Iba1 (gray) and DAPI (blue), boxed regions show an activated and phagocytotic microglia (a1, a3) in the impacted ipsilateral side, and ramified microglia at resting state (a2, a4) in the contralateral side. Scale bar in (A) 50 μm, in (a1-a6) 10 μm. (**B**) Quantification for the percentage of activated Iba1 positive cells (*n = 6* mice per group) in the impact side (ipsi) and contralateral side (contra) in sTBI and mTBI mice (see method). Dash line indicates the average value from the sham-operated group (*n = 5* mice). (**C**) Representative images of fluorescence immunostaining for GFAP (green) and DAPI (blue), boxed regions (c1-c4) show astrocytes in the impact side and the contralateral side. Scale bar in (C) 50 μm, in (c1-c6) 10 μm. (**D**) Quantification for the GFAP positive cells per mm^2^ (*n = 6* mice per group) in the impact side (ipsi) and contralateral side (contra) in sTBI and mTBI mice. Dash line indicates the average value from sham-operated group (*n = 5* mice). Data are presented as Mean ± SD; ***, *P* < 0.001; **, *P* < 0.01; *, *P* < 0.05; NS, non-significant (*P* > 0.05) by by Student’s t-test.


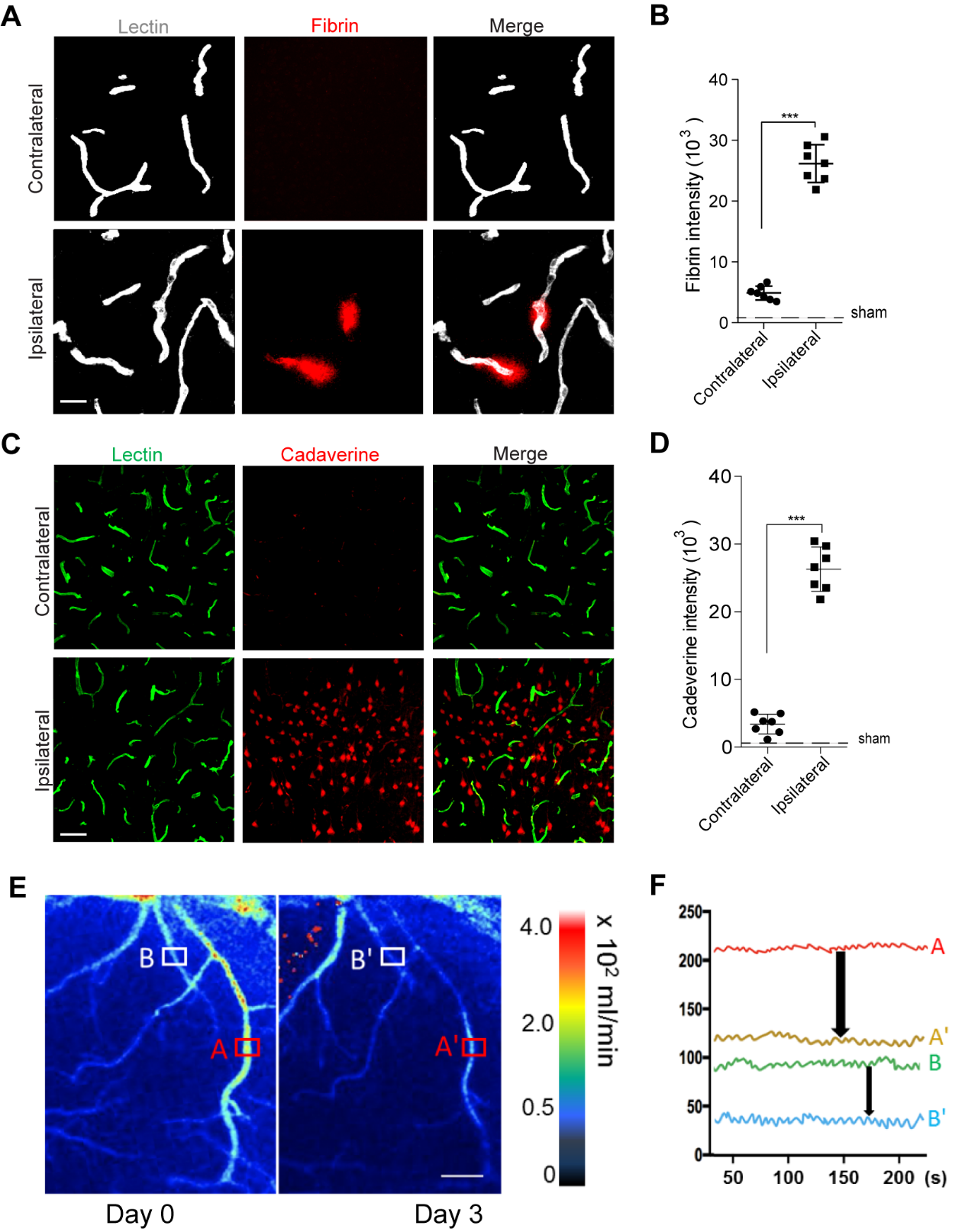


**Supplementary Fig. 4**. **Vascular impairment in the subacute phase of mTBI in mice.** (**A**-**B**) Representative images (**A**) and quantification (**B**) showing extravascular fibrin deposits in brain parenchyma 3 days after mTBI. Lectin (gray), fibrin (red). *n = 7* mice; Scale bar = 20 µm. (**C**-**D**) Representative images (**C**) and quantification (**D**) showing extravasation of intravenously administrated Alexa-555 cadaverine (red) in brain parenchyma 3 days after mTBI. Lectin (green), *n = 7* mice, scale bar = 50 µm. In B, D, data are presented as Mean ± SD; ***, P < 0.001 by Student’s t-test. Dash lines indicate average value from sham-operated group (*n = 3* mice). (**E**-**F**) Cerebral blood flow reduction 3 days after mTBI. Representative laser speckle contrast imaging (LSCI) images (**E**) and quantification of boxed regions (**F**) showing blood flow changes in the cortical regions 3 days after mTBI. Scale bar = 50 µm.


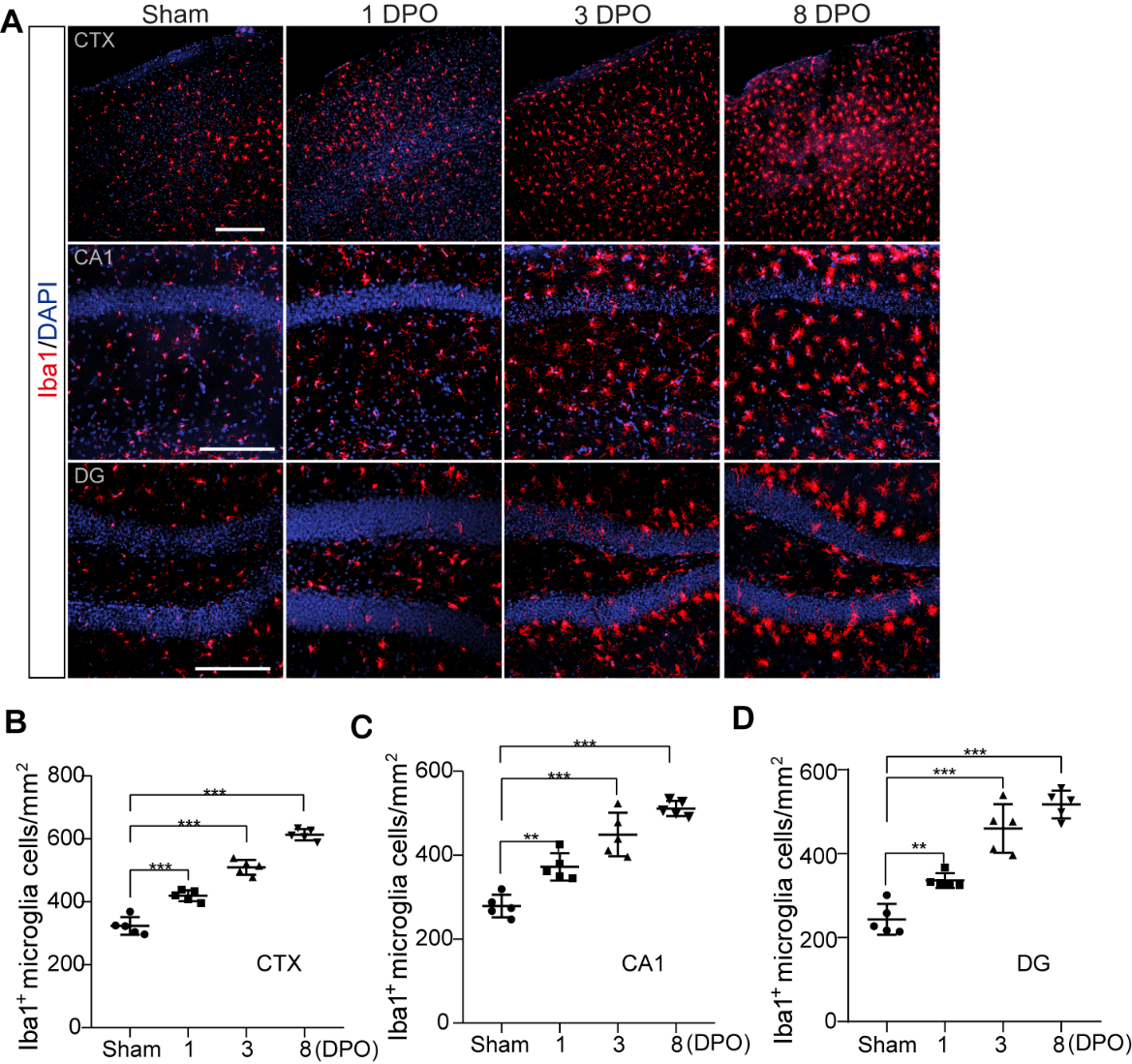


**Supplementary Fig. 5**. **The time course of microglial activation after mTBI in mice**. (**A**) Representative confocal microscope images showing Iba1-positive microglia cells (red) in cortex (CTX), Cornu Ammonis 1 (CA1) and dentate gyrus (DG) area 1-day, 3- and 8- days post operation (DPO). Scale bar = 100 µm. (**B**-**D**) Quantification for the Iba1-positive microglia cells per mm^2^ (*n = 5* mice per time point) in CTX, CA1 and DG area 1 day, 3- and 8-days post operation (DPO). Data are presented as Mean ± SD; ***, *P* < 0.001; **, *P* < 0.01; one-way ANOVA followed by Bonferroni’s post-hoc tests.
